# Annotations

**Published:** 1892-06-18

**Authors:** 


### Annotations.

**Lord Tennyson's Appeal.**

Lord Tennyson appealed to the public for £40,000 on behalf the Gordon Boys' Home. The public has responded with a tenth of that sum, £4,000, instead of £40,000. This result is both encouraging and discouraging. Discouraging to a certain extent to Lord Tennyson and the Gordon Boys' Home; but encouraging to charities generally, as showing that the public do not indiscriminately run after names however distinguished and great, but take the trouble to think for themselves, and to act accordingly. The Gordon Boys' Home is an entirely worthy institution, and it helps to keep green the memory of one of Britain's most heroic and single-minded sons. It is difficult to believe that anybody can wish it anything but success; and that success will most assuredly come if the institution is well and perseveringly managed. But it is evident that the success will by no means come *per saltum*; it must come by slow degrees, and in obedience to the laws of natural growth and development. Herein the responsible managers of old and well-tried charities, and, indeed, of all charities, may read an important lesson. The lesson is that charities must establish a well-defined character before they can reap the fruit which proved services merit. The way for hospitals and charities, as well as for private persons to win large and permanent success, is to constantly put into all their work more intelligence, more honesty, and more efficiency. When this is done, and done with constant persistence, nothing can prevent their complete and final success.

**The "Lancet's" Help.**

The *Lancet*, in an excellent supplement, 125,000 copies of which have been printed by the proprietors for gratuitous distribution, directs attention to three main points that bear upon the forthcoming Hospital Sunday collection. The first is the enormous growth of the population of London during the last twenty years, and the increased difficulty of supplying the vast numbers of helpless poor with adequate hospital accommodation; the second is an outline of the nature and extent of the work of hospitals during the past year; and the third an exposition of the beneficial influence exercised upon hospitals and charities generally by the judicious action of the council and members of the Hospital Sunday Fund. Our contemporary has had specially prepared a series of four small, but very instructive maps, which show not only the enlarging area of London, but the distribution and relative increase of density in the population of the various districts. The total increase of population in the county of London during the last twenty years has been 964,444. The centre of what we may call this enormous town tends to be depleted, whilst the suburbs, and more particularly some of them, show a congestion that constantly increases. The mere addition of population that has been made to London during the twenty years is, as the *Lancet* shows, equal to the whole population of Manchester, Leeds, and Norwich taken together. There is no difficulty in perceiving that if this enormously increased population is to be adequately supplied with hospital resources, a persistent effort of an unparalleled character must be made by every man, woman, and child who has the interest of the sick poor at heart. We have elsewhere dealt with representative portions of the work of the London hospitals during the past year, and on page 189 we give carefully collected and arranged statistics which deal with the hospital operations of the whole metropolitan area. We need not therefore further refer to this point here. But we take the opportunity of expressing the conviction that the *Lancet* has done well to direct attention to the entirely beneficial action of the Hospital Sunday Fund on the metropolitan charities and their resources during the whole period of the Fund's existence. The Hospital Sunday collection now adds the noble total of forty-five thousand pounds to the annual income of the hospitals, and this sum is steadily increasing. The *Lancet* shows that so far from the Sunday Fund's collections diminishing or drying up other sources of income, they have constantly stimulated benevolence on all hands. It could, indeed, hardly be otherwise. Nothing is so beneficial to a good cause as "bold advertisement"; and what other advertisement could be at once so bold and so universally effective as the preaching of specially prepared and specially announced sermons on a specially set-apart Sunday, in every cathedral, church, chapel, and meeting-house in every part of this vast London? It cannot be doubted that the Hospital Sunday Fund has poured new and vivifying life-blood into every artery and vein of the whole hospital organism. We look forward to the collection of the present year, not only without anxiety, but with animated hope. And even if the collection should be less full and generous than we anticipate we shall be in no wise dismayed. The Hospital Sunday Fund, and the whole charitable world of which it is so natural and fitting a part, has before it a future which can never be anything but great and growing so long as Britain continues to march in the van of virtue and of nations.

**The Street Beggars of Charity.**

It is an old saying that when things get to a certain pitch of badness comes the mending. The case of the girl, Maud Taylor, charged with gaining money under false pretences by means of a collecting box, professedly belonging to St. Mary's Hospital, seems to show that the mending point in respect to street collections for charities has been reached. The protest against such a form of obtaining contributions made by the secretary of St. Mary's Hospital is almost universally endorsed by the managers and friends of charities generally. It must be obvious to all that street collection is most distasteful to the public. The same person may be worried by a dozen collectors before he has left his doorstep half-an-hour, and the irritation caused by these reiterated solicitations is enough to make the very name of "charity" hateful. It is argued by some that this method forms the only means of reaching a large class of people who do not contribute through church collections or otherwise towards our charities. Supposing this to be the case, we still hold that the end does not justify the means, nor is the result at all adequate to the outlay. Experience goes to show that the sums collected are so small as to be insignificant when the expenses are deducted. Most of the contributors to such boxes, if reached in some other manner, would double or treble the small sum they frequently pay for the purpose of escaping further annoyance. Under the cloak of charity much evil is sometimes effected, for the instruments are but human, and the term in its widest sense is little understood. For this and other reasons it must be clear to all that the time has come when effectual means should be taken to prevent these collections in the public streets from proving a source of temptation, and the hospital authorities would be well advised to concert measures with the police to effectually put a stop to this dangerous nuisance. Hawking boxes in the streets should be made illegal and be punishable by law.

